# Comparison of Mercury Distribution Between Liver and Musc - A Biomonitoring of Fish from Lightly and Heavily Contaminated Localities

**DOI:** 10.3390/s8074095

**Published:** 2008-07-10

**Authors:** Marcela Havelková, Ladislav Dušek, Danka Némethová, Gorzyslaw Poleszczuk, Zdeňka Svobodová

**Affiliations:** 1 University of Veterinary and Pharmaceutical Sciences, Faculty of Veterinary Hygiene and Ecology Department of Public Health and Toxicology, Palackého 1-3, 612 42, Brno, Czech Republic; 2 Masaryk University, Faculty of Medicine and Faculty of Science, Institute of Biostatistics and Analyses, Kamenice 126/3, 625 00 Brno, Czech Republic; 3 Szczecin University, Faculty of Natural Sciences, Chair of Chemistry, ul. Felczaka 3 A, 71-412 Szczecin, Poland; 4 University of South Bohemia in České Budějovice, Research Institute of Fish Culture and Hydrobiology, 389 25 Vodňany, Czech Republic

**Keywords:** Hg liver/muscle ratio, indicator fish, predator, non-predator, river contamination

## Abstract

Tissue samples from 1,117 fish of 25 species were collected from 1991 through 1996 at 13 locations along the River Elbe. The principal indicator species were perch (*Perca fluviatilis*) (n=118), chub (*Leuciscus cephalus* L.) (n=113) and roach (*Rutilus rutilus*) (n=138). Mercury (Hg) concentrations in muscle and liver were determined by atomic absorption spectrometry. The liver/muscle index in three indicator species from heavily contaminated and lightly contaminated localities were significantly different. In fish from heavily contaminated localities, Hg was deposited preferentially in the liver (the depository for inorganic and organic forms of Hg), while in lightly contaminated areas, it was deposited preferentially in muscle.

## 1. Introduction

Mercury (Hg) belongs to major pollutants of the aquatic environment. Because of the extreme toxicity of its organic forms, its ability to bioaccumulate in aquatic organisms and its long-term persistence in sediments, mercury concentration in the environment needs to be closely monitored.

Although it is not a biogenic element in living organisms, Hg nevertheless accumulates in certain tissues. The highest Hg accumulations exist in aquatic organisms, specifically in fish. When it enters the aquatic environment, Hg is usually in its inorganic form and is transformed into the much more harmful, organic Hg, through the process of methylation. The concentration and type of Hg also depends on the character of the sediment [1]. The association between concentrations of inorganic Hg in tissues and in sediments has been demonstrated [2, 3].

Metals are transferred from sediments to the food chain. The amount of Hg in the organism is affected by its position in the food chain [4-6], its size, age [2, 5, 7, 8] and duration of exposure [9]. There is also an association between Hg concentrations and fish weight [10, 11].

The main pathway for inorganic Hg intake into fish is the digestive tract, but other pathways are the skin and gills. Mercury is transported within the organism bound to blood plasma proteins. The liver, as the organ that participates in redistribution, detoxification and transformation of pollutants, is the target for inorganic Hg [8, 12]. Organic Hg de-methylated to its inorganic form in the liver.

Some authors believe that Hg distribution in fish tissues from heavily contaminated and lightly contaminated localities is different [10, 12, 13-16]. This was not corroborated by Wang et al. (2005) [17] in their study on frogs (*Rana chensinensis*), or by Honda et al. (1983) [9] or Chen et al. (2004) [18].

The aim of this study was to compare the distribution of Hg in fish tissues from heavily and lightly contaminated localities. The comparison was based on Hg concentrations in fish collected between 1991 and 1996 from several localities along the River Elbe as part of the “Elbe Project”.

The River Elbe is one of the most extensive aquatic ecosystems in Central Europe. It is 1,091 km long (370.2 km of which are in the Czech Republic). Its extensive basin, an area of 148 268 km^2^, lies within the boundaries of two countries, the Czech Republic and Germany. Pollution of the Elbe River originated mainly from inflow of water from catchment areas contaminated by municipal wastes and industrial discharges (chemical industries, paper mills, waste water works, shipbuilding yards) [19] and from tributaries of Elbe [20]. Thus sites located downstream of large cities along the river are the most representative models for long-term monitoring surveys and for the determination of levels of contamination. Significant sources of contamination, besides industrial and municipal waste, include agriculture, uncontrolled erosion, soil leaching and surface runoff.

## 2. Materials and methods

**Figure 1.**
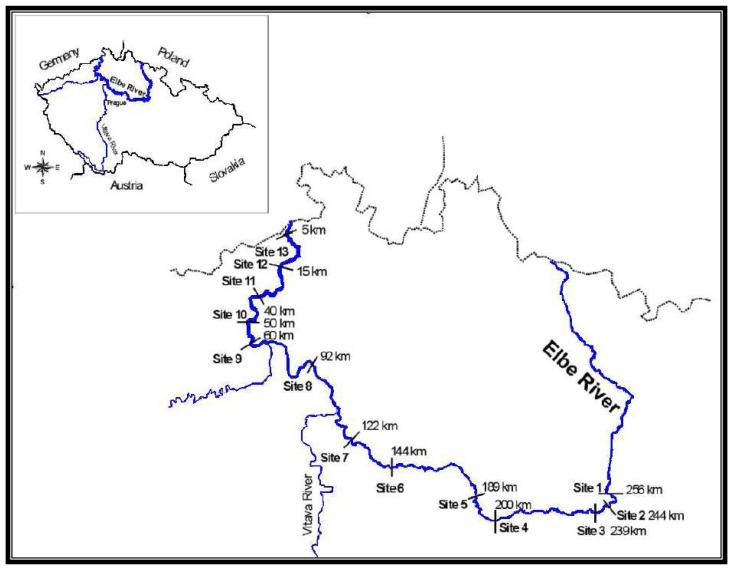
Geographical location of the sites (Czech Republic). The number of fish captured at individual sites is given in parenthesis. Site 1 - Opatovice downstream (n = 120), Site 2 - Pardubice upstream (n = 98), Site 3 -Pardubice downstream (n = 65), Site 4 - Kolín upstream (n = 48), Site 5 - Kolín downstream (n = 72), Site 6 - Čelákovice downstream (n = 69), Site 7 - Neratovice downstream (n = 77), Site 8 - Štětí downstream (n = 30), Site 9 - Lovosice downstream (n = 75), Site 10 - Variov downstream (n = 25), Site 11 - Ústí nad Labem downstream (n = 93), Site 12 - Decín downstream (n = 279), Site 13 - Hfensko downstream (n = 66;.

Fish tissue samples were collected between 1991 and 1996 at 13 sites along the River Elbe. The location and description of individual sites, and the number of fish collected at each site are given in Figure 1.

Lightly contaminated localities included Opatovice (typical lightly contaminated locality without significant anthropogenic influence) and localities upstream and downstream of the city of Pardubice (background sites). Heavily contaminated sites included areas upstream and downstream of the cities of Kolín (industrial and municipal waste), Čelákovice, Neratovice (chemical production), Štětí (paper mills), Lovosice (chemical industry), Variov (ship-building yard, docks), Ústí nad Labem (municipal waste, chemical industry, organic waste discharges), Decín (municipal waste, chemical industry), and Hfensko (municipal waste, chemical industry; [6].

### 2.1. Collection of fish samples

Altogether 1,117 fish of 25 species were captured by electrofishing. All fish were captured in summer (from June to August). The fish species examined and their feeding habits are shown in Table 1. Fish were weighed and measured upon capture and their ages determined by scale analysis. Samples of muscle and liver were removed, placed in polyethylene bags, labelled, and transported in cooled containers to a freezer where they were stored at -18°C.

## 2.2. Total mercury determination

Total Hg tissue concentrations were determined by the AMA 254 single-purpose analyzer, which is based on combustion-amalgamation atomic absorption. No chemical pre-treatment of the samples was needed. A sample of fish tissue (liver or muscle) of known weight was placed on a sampling boat. By controlled heat, the sample was first dried and then thermally decomposed. The decomposition products were carried by oxygen flow to the second, catalytic section, of the instrument. The further decomposition products were then carried to an amalgamator which selectively traps mercury. Detection limit of Hg in the samples is 0.001 mg kg^-1^. Concentration of mercury in fish tissue is reported in terms of wet weight. Recovery of the method was 82 ± 6%. Mercury liver/muscle index was calculated for only 922 fish, because this was the number from which both liver and muscle were removed.

Concentrations of Hg in water along the River Elbe were also determined. No significant differences were found in concentrations of Hg in water between 1991 and 1996 at monitored localities.

### 2.3. Statistical analysis

Distribution of each of the fish species in lightly and heavily contaminated localities was tested by mean of X^2^ goodness-of-fit test. Only 15 species whose occurrence in lightly and heavily contaminated localities did not differ significantly were included in further analyses (Appendix 1 – Table 5).

**Figure 2.**
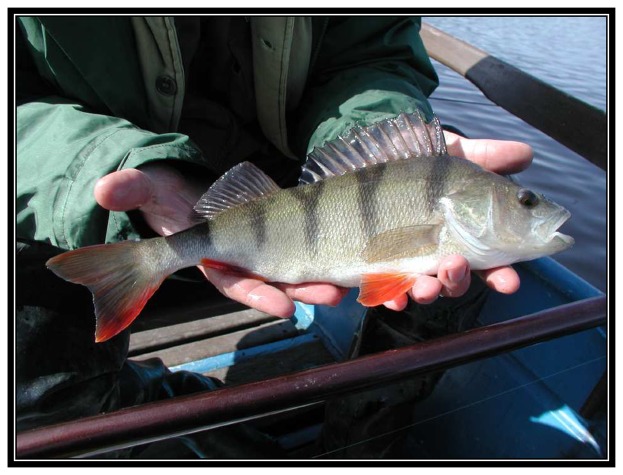
The main indicator species – Perch (*Perca fluviatilis*).

**Figure 3.**
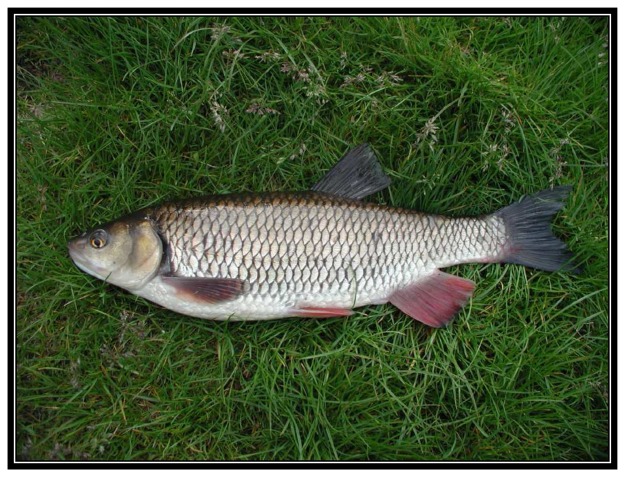
The main indicator species – Chub (*Leuciscus cephalus* L.).

**Figure 4.**
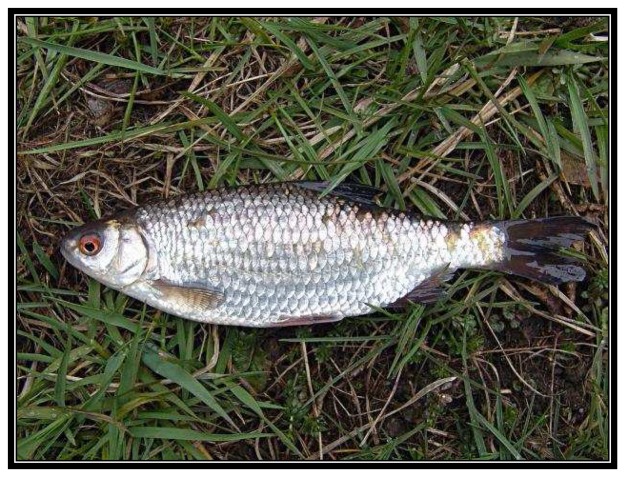
The main indicator species – Roach (*Rutilus rutilus)*.

To control for the affect of fish age on mercury concentration, linear regression was performed on each of the 15 species and mercury parameter (Appendix 2 - Table 6). The independent variable in the regression was fish age. The dependent variables were mercury concentration in liver, mercury concentration in muscle, and liver/muscle index. Regression residuals from linear regressions for each species and mercury parameter were saved. The values of these residuals were compared between heavily and lightly contaminated localities as well as between liver and muscle, considering only the mercury concentration which is not explained by fish age. Three largest indicator species groups (perch *Perca fluviatilis*, n = 118; chub *Leuciscus cephalus*, n = 113; roach *Rutilus rutilus*, n = 138) (Figures 2, 3 and 4) and groups comprising predators and non-predators were analysed.

The Kolmogorov-Smirnov test was used for assessing the normal distribution of residuals in perch, chub, roach, predator and non-predator in heavily and lightly contaminated localities. Almost all tests resulted in non-normal distribution of residuals in both heavily and lightly contaminated localities (P < 0.05). This holds true for residuals of mercury concentration in liver and in muscle, as well as liver/muscle index. Therefore non-parametric tests were used to analyse the data. To compare values in heavily and lightly contaminated locations, the Mann-Whitney U test was used. A comparison between liver Hg levels and muscle Hg levels in fish from lightly as well as from heavily contaminated locations was performed using the Wilcoxon matched pairs test.

## 3. Results

To compare Hg levels in fish tissues from heavily contaminated and lightly contaminated localities, the liver/muscle index was used. The liver/muscle index is ratio of liver to muscle Hg concentrations [Hg liver (μg g^-1^)/Hg muscle (μg g^-1^)]. Mercury liver/muscle index adjusted for fish age, for three indicator fish species from heavily and lightly contaminated localities (perch, chub and roach) are given in Table 2. All of the ratios residuals were significantly higher (P < 0.001; Table 2) in fish from heavily contaminated localities than from lightly contaminated localities. Mercury concentration in muscle was higher than in liver of three indicator fish species from lightly contaminated sites (Wilcoxon matched pairs test: perch: n = 32; P < 0.001; chub: n = 29; P < 0.001; roach: n = 32; P < 0.001). In heavily contaminated localities, Hg concentration in liver was higher than that in muscle, although the difference was statistically significant only in perch (Wilcoxon matched pairs test: perch: n = 71; P = 0.012; chub: n = 82; P = 0.272; roach: n = 90; P = 0.360). Differences in liver/muscle index (adjusted for age) were also found in predatory fish (n = 208; U = 1192; P < 0.001) and non-predatory fish (n = 428; U = 3931; P < 0.001) when heavily and lightly contaminated localities were compared. The ratio residual for predatory fish from heavily contaminated localities (0.055) was higher than for non-predatory species (0.028), although the difference was not statistically significant (n = 473; U = 24735; P = 0.828). In lightly contaminated localities, the ratio residual in predatory fish was slightly, but not significantly, higher (-0.506) than in the non-predatory species (-0.581) (n = 163; U = 2488; P = 0.322). Mercury concentrations in liver and muscle change with the level of environmental contamination, and consequently the ratios change.

Mercury concentration, adjusted for age, in muscle and liver of three species of indicator fish from heavily and lightly contaminated localities are given in Tables 3 and 4. The highest concentrations of Hg were found in perch, the representative of predatory fish. Mercury content in muscle in the three indicator fish species differed significantly between heavily and lightly contaminated localities (in all three species P < 0.001; Table 3), being higher in heavily contaminated localities. The same holds true for liver Hg concentration residuals (in all three species P < 0.001; Table 4). A comparison among residuals of concentrations of Hg in liver and muscle of predatory and non-predatory fish species from heavily and lightly contaminated localities showed that the highest Hg concentrations were in the liver of predatory fish species from heavily contaminated localities (0.063 μg g^-1^). The lowest Hg concentrations were found in the liver of predatory fish from lightly contaminated localities (-0.453 μg g^-1^). In heavily contaminated localities, the residuals of muscle Hg concentrations were higher in predatory species than in non-predatory species. However, the difference was not significant (n = 536; U = 30856; P = 0.278). On the other hand, the difference was significant in lightly contaminated localities (n = 163; U = 1413; P < 0.001). Similar results were also found in the liver. Residuals of liver Hg concentrations were higher in predatory than in non-predatory fish. The difference was not significant in heavily contaminated localities (n = 474; U = 23017; P = 0.136), but was significant in lightly contaminated localities (n = 163; U = 1773; P < 0.001).

Distribution of fish species in heavily and lightly contaminated localities and regression equations of effect of age on mercury concentration in muscle, liver and liver and muscle mercury concentration ratio are shown in Appendix 1 (Table 5) and Appendix 2 (Table 6).

## 4. Discussion

A comparison between Hg concentrations in tissues of fish from heavily contaminated and lightly contaminated localities showed the existence of differing mercury distribution in fish from those localities. In all three indicator fish species, the liver/muscle index was significantly higher (Table 2) in fish from heavily contaminated localities than in fish from lightly contaminated localities. While the target organ for Hg accumulation in fish from heavily contaminated localities was the liver, the main target organ for Hg accumulation in fish from lightly contaminated localities was muscle.

The distribution of mercury in muscles and internal organs of fish depends, *inter alia*, on the degree of contamination of the environment [10, 21]. The liver was selected for analysis because it is a good indicator of environmental pollution. The liver has the ability to accumulate large quantities of pollutants from the external environment, and also plays an important role in storage, redistribution, detoxification, and transformation of pollutants [22]. Higher Hg concentration in liver compared with that in muscle has been corroborated by Kennedy (2003) [15] and Gonzalez et al. (2005) [16], who exposed fish (common goldfish, *Carassius auratus* and zebrafish, *Danio rerio*, respectively) to various Hg concentrations. Data from the literature indicate that when Hg concentrations in fish muscle are low (below approximately 0.5 μg g^-1^), Hg concentration in muscle is about twice that in liver. When higher muscle concentrations of Hg are reached (> 1 μg g^-1^), the ratio is reversed, and Hg concentrations in the liver will be several times higher than that in muscle [23].

In Hg-polluted locations, Hg concentrations in internal organs are usually significantly higher than Hg concentrations in muscle [10, 24]. In their study of sea bass (*Dicentrarchus labrax*) from heavily contaminated localities, Abreu et al. (2000) [10] found up to twice the Hg concentration in the liver as in muscle.

The fact that Hg concentration in muscle of fish captured from lightly contaminated localities is usually higher than that found in their internal organs (liver, kidney) has been reported in studies of common carp (*Cyprinus carpio*) [12], seven species of fish from the Skalka reservoir [24], pike-perch (*Stizostedion lucioperca* L.) and bream (*Abramis brama*) from Lake Balaton in Hungary [14], tusks (*Brosme brosme*) captured off the coastline (a lightly contaminated locality) [13], and *Odontotesthes microlepidotus* from lightly contaminated localities [25]. Mercury distribution in lightly contaminated localities seems to take the following pattern: muscle > kidney > liver > gonads [26, 27]. Higher Hg concentrations in muscle compared to liver have been reported in fish from Otradovice, a lightly contaminated locality in the River Jizera [28]; in tissue of fish from some selected lightly contaminated ponds studied for metal concentrations in tissues [29]; and in European eel *(Anguilla anguilla*) and brown trout (*Salmo trutta*) from the River Ferrerias in Spain (a lightly contaminated locality) [30].

In their study on *Rana Chensinensis* from both heavily contaminated localities and lightly contaminated localities, Wang et al. (2005) [17], on the other hand, demonstrated an average of 50% higher Hg concentration in the liver than in muscle. Honda et al. (1983) [9] found Hg concentrations in liver to be twice that in muscle in *Pagothenia borchgreinki* from the Antarctic, an area free of any significant anthropogenic pollution with heavy metals. Similar conclusions have been drawn by Chen et al. (2004) [18], who measured tissue Hg concentration in localities with different levels of contamination. In most cases, liver Hg concentrations were higher than muscle Hg concentrations irrespective of the degree to which the location was polluted.

Mercury concentrations in fish tissues from heavily and lightly contaminated localities differed in accordance with feeding habits of individual species. Mercury concentrations in predatory fish tissues were significantly higher than those of non-predatory fish (P < 0.001). The amount of Hg accumulated in fish tissues is related to their position in the food chain. Older predatory fish, as the end link of the food chain, show higher Hg concentrations than non-predatory fish [6, 8]. Also, the diet of predatory fish is richer in lipids, giving the liver a greater capacity for storing lipid-soluble methylmercury than that of non-predatory fish. Piscivores tend to have a higher liver/muscle index compared with non-piscivorous species. In nonpiscivores, the liver/muscle index is approximately one-to-two, while in piscivores the ratio is near one-to-one [23].

Mercury occurs in two basic forms in fish tissues, the inorganic form and the organic form, methylmercury. The two forms of Hg differ in concentration and distribution in the fish body. Methylmercury is preferentially distributed to muscle, where it binds to protein-rich cystein (in sarcoplasmatic proteins). Methylmercury concentration in muscle follows total Hg concentrations, and the methylmercury to total Hg ratio in muscle usually exceeds 80% [1]. Thus in muscle, Hg occurs mostly as its organic form, in contrast to the liver, where accumulation is mostly of the inorganic [8, 12, 24, 31-34].

## 5. Conclusion

In conclusion, the liver is the organ where de-methylation of the organic form of Hg to the less toxic inorganic form takes place [35], and where the latter is stored and metabolized. The methylmercury to total Hg ratio in the liver is lower than that in muscle. A comparison between Hg concentrations in tissues showed the existence of differing Hg distributions in fish from heavily contaminated and lightly contaminated localities. These results indicate that fish are able to tolerate low Hg concentrations. If Hg concentrations in tissues exceed 1 μg g^-1^ Hg is redistributed from muscle, which leads to an increase of Hg concentration in the liver.

## Figures and Tables

**Table 1.** Examined fish species and their feeding habits.

Fish species	Common name	Feeding guild
*Abramis brama*	Bream	Benthophagous
*Alburnoides bipunctatus*	Spirlin, riffle minnow	Planctivorous
*Alburnus alburnus*	Bleak	Planctivorous
*Anguilla anguilla*	European eel	Predator
*Aspius aspius*	Asp	Predator
*Barbus barbus*	Barbel	Benthophagous
*Blicca bjoerkna*	White bream, silver bream	Benthophagous
*Carassius auratus*	Gibel carp, goldfish	Planctivorous
*Esox lucius*	Pike	Predator
*Gobio gobio*	Gudgeon	Benthophagous
*Gymnocephalus cernuus*	Ruffe, pope	Benthophagous
*Ictalurus nebulosus*	Catfish, brown bullhead	Benthophagous
*Leuciscus cephalus*	Chub	Omnivorous
*Leuciscus idus*	Ide, orfe	Omnivorous
*Leuciscus leuciscus*	Dace	Omnivorous
*Oncorhynchus mykiss*	Rainbow trout	Predator
*Perca fluviatilis*	Perch	Predator
*Rutilus rutilus*	Roach	Benthophagous
*Salmo trutta*	Trout	Predator
*Scardinius erythrophthalmus*	Rudd	Phytophagous
*Silurus glanis*	Wels, sheatfish	Predator
*Stizostedion lucioperca*	Pikeperch, zander	Predator
*Tinca tinca*	Tench	Benthophagous
*Thymallus thymallus*	Grayling	Benthophagous
*Vimba vimba*	Vimba bream	Benthophagous

**Table 2.** Liver/ muscle index in three indicator fish species, predators and non-predators, from heavily (HC) and lightly contaminated (LC) localities (effect of age subtracted).

Fish species	Locality contamination	N	Mean	Median	Minimum	Maximum	Std.Dev.	Mann-Whitney U test
PERCH	HC	71	0.202	0.139	-0.791	1.514	0.549	U = 268
	LC	32	-0.448	-0.554	-0.892	2.170	0.535	P < 0.001

CHUB	HC	82	0.148	0.068	-0.553	2.537	0.488	U = 230
	LC	29	-0.420	-0.487	-0.783	0.835	0.320	P < 0.001

ROACH	HC	90	0.242	-0.105	-0.738	5.669	1.075	U = 187
	LC	32	-0.680	-0.721	-1.002	-0.005	0.197	P < 0.001

PREDATOR	HC	160	0.154	0.055	-1.592	3.190	0.687	U = 1192
	LC	48	-0.512	-0.506	-1.658	2.170	0.542	P < 0.001

NO PREDATOR	HC	313	0.217	0.028	-0.963	5.669	0.812	U = 3931
	LC	115	-0.590	-0.581	-1.959	1.189	0.409	P < 0.001

**Table 3.** Muscle concentration (μgg-1) in three indicator fish species, predators and non-predators, from heavily (HC) and lightly contaminated (LC) localities (effect of age subtracted).

Fish species	Locality contamination	N	Mean	Median	Minimum	Maximum	Std.Dev.	Mann-Whitney U test
PERCH	HC	86	0.152	0.043	-0.842	3.941	0.647	U = 351
	LC	32	-0.407	-0.370	-0.820	-0.038	0.213	P < 0.001

CHUB	HC	84	0.142	-0.100	-0.365	2.564	0.554	U = 110
	LC	29	-0.412	-0.432	-0.558	-0.069	0.127	P < 0.001

ROACH	HC	104	0.062	0.014	-0.207	1.180	0.198	U = 54.5
	LC	32	-0.200	-0.192	-0.308	-0.094	0.042	P < 0.001

PREDATOR	HC	188	0.107	0.060	-0.842	3.941	0.481	U = 705
	LC	48	-0.417	-0.370	-1.030	-0.038	0.202	P < 0.001

NO PREDATOR	HC	348	0.087	0.012	-0.558	2.564	0.352	U = 3627.5
	LC	115	-0.264	-0.228	-0.558	0.024	0.140	P < 0.001

**Table 4.** Liver concentration (μgg^-1^) in three indicator fish species, predators and non-predators, from heavily (HC) and lightly contaminated (LC) localities (effect of age subtracted).

Fish species	Locality contamination n	N	Mean	Median	Minimum	Maximum	Std.Dev.	Mann-Whitney U test
PERCH	HC	71	0.225	0.113	-0.973	2.028	0.549	U = 166
	LC	32	-0.500	-0.440	-0.899	-0.075	0.210	P < 0.001

CHUB	HC	82	0.182	-0.059	-0.546	3.950	0.794	U = 158
	LC	29	-0.515	-0.523	-0.764	-0.209	0.168	P < 0.001

ROACH	HC	91	0.123	-0.037	-0.323	2.669	0.473	U = 46
	LC	32	-0.349	-0.341	-0.473	-0.239	0.062	P < 0.001

PREDATOR	HC	160	0.170	0.063	-1.283	3.379	0.679	U = 830
	LC	48	-0.566	-0.453	-1.846	-0.075	0.295	P < 0.001

NO PREDATOR	HC	314	0.158	-0.031	-0.684	3.950	0.605	U = 2939
	LC	115	-0.431	-0.369	-1.278	0.015	0.222	P < 0.001

## Appendix 1

**Table:** (Table 5) Distribution of fish species in heavily and lightly contaminated localities. Occurence of species marked with asterisk in lightly and heavily contaminated localities differed significantly. (N - number of individuals; X2-goodness-of-fit test used)

**Fish species**	**N**	**X^2^**	**df**	**Significance level**
Abramis brama *	137	10.203	1	**P = 0.001**
Alburnus alburnus *	73	6.662	1	**P = 0.010**
Anguilla anguilla	30	2.602	1	P = 0.107
Aspius aspius	21	3.368	1	P = 0.066
Barbus barbus	32	1.749	1	P = 0.186
Blicca bjoercna	66	3.289	1	P = 0.070
Carassius auratus	27	1.749	1	P = 0.186
Cyprinus carpio *	27	14.479	1	**P < 0.001**
Esox lucius *	49	4.631	1	**P = 0.031**
Gobio gobio *	60	35.875	1	**P < 0.001**
Gymnocephalus cernua	4	2.321	1	P = 0.128
Ictalurus nebulosus *	35	7.185	1	**P = 0.007**
Leuciscus cephalus	113	0.019	1	P = 0.892
Leuciscus idus	38	0.045	1	P = 0.833
Leuciscus leuciscus	10	5.820	1	P = 0.016
Perca fluviatilis	118	0.272	1	P = 0.602
Rutilus rutilus	138	0.266	1	P = 0.606
Salmo trutta	5	2.321	1	P = 0.128
Scardinius erythrophthalmus *	29	15.030	1	**P < 0.001**
Stizostedion lucioperca	63	3.343	1	P = 0.067
Tinca tinca	29	1.292	1	P = 0.256
Vimba vimba	8	0.600	1	P = 0.439

Lota lota	2			
Silurus glanis	2			
Thymallus thymallus	1			

Predator vs. no predator	702	1.759	1	P = 0.185

## Appendix 2

**Table:** (Table 6) Regression equations of effect of age on mercury concentration in muscle, liver and liver and muscle mercury concentration ratio. (Fish species with different distribution in heavily and lightly contaminated localities were not included.)

	**Hg in muscle**				**Hg in liver**				**Hg in liver / Hg in muscle**			
	**intercept**	**slope**	**r^2^**	**P**	**intercept**	**slope**	**r^2^**	**P**	**intercept**	**slope**	**r^2^**	**P**
Anguilla anguilla	0.526	-0.010	0.004	0.726	1.085	-0.012	0.001	0.911	1.674	0.037	0.002	0.825
Aspius aspius	0.491	0.135	0.197	0.044	2.120	-0.021	0.001	0.923	3.027	-0.242	0.096	0.196
Barbus barbus	0.233	0.070	0.036	0.296	0.089	0.077	0.038	0.287	0.344	0.073	0.058	0.183
Blicca bjoercna	0.358	0.006	0.003	0.689	-0.002	0.118	0.089	0.025	0.569	0.147	0.064	0.060
Carassius auratus	0.173	0.016	0.105	0.099	0.088	0.004	0.016	0.534	0.489	-0.005	0.002	0.835
Gymnocephalus cernua	0.212	-0.011	0.228	0.523								
Leuciscus cephalus	0.308	0.032	0.018	0.161	0.058	0.080	0.051	0.017	0.418	0.071	0.085	0.002
Leuciscus idus	0.350	-0.011	0.013	0.493	0.458	-0.004	0.000	0.931	0.605	0.106	0.032	0.294
Leuciscus leuciscus	0.229	0.042	0.447	0.035	0.283	0.008	0.006	0.839	1.045	-0.062	0.051	0.531
Perca fluviatilis	-0.147	0.335	0.254	< 0.001	-0.226	0.379	0.335	< 0.001	0.925	0.056	0.009	0.328
Rutilus rutilus	0.236	0.015	0.018	0.116	0.255	0.028	0.013	0.218	0.855	0.048	0.007	0.346
Salmo trutta	0.289	-0.082	0.435	0.341								
Stizostedion lucioperca	0.710	-0.023	0.010	0.427	0.583	-0.005	0.000	0.913	0.881	-0.014	0.002	0.708
Tinca tinca	0.692	-0.047	0.106	0.085	0.434	-0.021	0.010	0.625	0.656	-0.017	0.007	0.693
Vimba vimba	0.746	-0.052	0.030	0.682	0.177	0.124	0.016	0.763	-1.050	0.492	0.132	0.377
